# Nativity and racial/ethnic differences in HPV infection among U.S. adults, NHANES 2007–2016

**DOI:** 10.3389/fpubh.2025.1604954

**Published:** 2025-09-10

**Authors:** Leslie E. Cofie, Lei Xu, Breanna Alligood, Nikhil Bhagat, Sarah Maness, Mi Hwa Lee, Alice Richman

**Affiliations:** ^1^Department of Health Education and Promotion, East Carolina University, Greenville, NC, United States; ^2^Physician Assistant Studies Program, Weill Cornell Medicine, New York, NY, United States; ^3^Celia Scott Weatherhead School of Public Health and Tropical Medicine, Tulane University, New Orleans, LA, United States; ^4^School of Social Work, East Carolina University, Greenville, NC, United States

**Keywords:** HPV infection, HPV vaccination, foreign-born individuals, race/ethnicity, national health surveillance

## Abstract

**Introduction:**

Although Human Papillomavirus (HPV) is the most common sexually transmitted infection globally, data on the rate of infection among immigrant populations in the U.S. is limited This study examined changes in HPV infection prevalence overtime by foreign-born status, sex, and race/ethnicity, as well as race/ethnic differences in HPV infection among foreign-born individuals.

**Methods:**

The 2007–2016 National Health and Nutrition Examination Survey (NHANES) data on adults, 18–34 years (N = 4,523) were analyzed. The outcome measures included any HPV infection and vaccine-type infection. Independent measures were foreign-birth status, sex, and race/ethnicity. Differences in HPV infection prevalence were assessed using chi-square test, and multivariate binary logistic regression analyses were used to examine the relationship between race/ethnicity and HPV infection among foreign-born individuals.

**Results:**

Overall prevalence of any HPV infection was 43% and vaccine-type HPV infection was 8%. Foreign-born individuals had significantly lower HPV infection, vaccine-type infection, and vaccination initiation rates compared to US-born adults (p < 0.001). Among foreign-born adults, the odds of HPV infection were higher for Black individuals compared to White individuals (aOR = 3.38; 95% CI: 1.3–8.6), after adjusting for survey cycle, sociodemographic covariates and sexual partners; and also higher among Hispanic females (aOR = 2.99; 95% CI: 1.26–7.11) and Black females (aOR = 5.27; 95% CI: 1.51–18.41) compared to White females. No significant associations were observed among foreign-born males.

**Discussion:**

The implementation of public health measures to increase vaccination rates, which can effectively prevent HPV infections, should target foreign-born Black and Hispanic adults.

## Introduction

Human papillomavirus (HPV) is the most common sexually transmitted infection worldwide (1). While some HPV infections can persist for years with no symptoms, certain types of HPV infection can cause cancer, including cervical, penile, vaginal, vulvar, anal and oropharyngeal (2). Globally, HPV infections account for nearly all cervical cancers and HPV types 16 and 18 account for 70% of cervical cancers (2, 3). Within the United States alone, between 2015–2019 about 36,500 cancers were attributed to at least one type of HPV infection (4). The primary solution to protecting the U.S. population from HPV infection has been HPV vaccination (5). The National Advisory Committee on Immunization Practices recommends HPV vaccine to both male and female adolescents and young adults, ages 11–26 years; but HPV vaccination can start as early as 9 years old (6). Also, adults ages 27–45 may be vaccinated through shared clinical decision making with their providers (6). Although HPV vaccination is proven to be safe, effective, and provides long-lasting protection against HPV infection, vaccine coverage remains lower than the Healthy People 2030 goal of 80%. In 2022, for example, only 62.6% of adolescents 13–17 and 47.4% of young adults aged 18–26 reported HPV vaccine uptake (7).

Since increasing HPV vaccine uptake is the ultimate solution for infection reduction and subsequent cancer prevention, monitoring population differences in HPV infections allows the U.S. to identify high-risk groups that can greatly benefit from HPV vaccination. Disparities in HPV infections are often attributed to factors such as age, gender, race/ethnicity, and having multiple sexual partners (8–11). Studies assessing sex and racial/ethnic differences in HPV infections revealed higher rates of infection among men and racial/ethnic minorities (11–13). Furthermore, available research data suggests that HPV infections are lower among foreign-born individuals compared with those born in U.S (14, 15). These findings are limited in that they are either from less recent national health surveillance data, include only female participants, or do not examine HPV infection differences among foreign-born sub-populations.

There is an urgent need to monitor national level prevalence of HPV infection by nativity status, as the percentage of foreign-born populations in the U.S. increased from 4.7% in 1970 to approximately 14% in 2022 (16). Moreover, many of these immigrants come from developing regions, e.g., Asia and Latin America, that have high burdens of HPV-associated cancers (17). Few studies have leveraged the availability of multiple years of national level health survey data to examine trends in HPV infections among foreign-born populations. Whereas numerous studies have examined population differences in HPV infections – including race/ethnicity and sex – overtime, additional research is needed to understand how nativity status further impacts these differences. Monitoring these trends helps to identify points of intervention and assess the progress of ongoing efforts to reduce inequities in HPV-associated infections and cancer burdens among U.S.- and foreign-born adults (18). In this study we utilized the National Health and Nutrition Examination Survey to (1) assess trends in HPV infections by foreign-born status, sex, and race/ethnicity across multiple survey cycles; and (2) examine the association between race/ethnicity and HPV infection among foreign-born individuals.

## Methods

### Study design and data

Data for this study is from the National Health and Nutrition Examination Survey (NHANES), which is conducted by the Center for Disease Control and Prevention’s (CDC) National Center for Health Statistics (NCHS). These ongoing cross-sectional surveys of a representative sample of the non-institutionalized U.S. civilian population examine health and nutritional status, and prevalence of major diseases and associated risk factors. Detailed description of the NHANES is provided elsewhere (19). Briefly, over two-year periods, the NCHS uses a complex, stratified, multistage probability sampling approach to recruit participants to take part in household interviews (using audio computer-assisted self-interviewing software) and physical examination by trained personnel in a mobile examination center. The East Carolina University Institutional Review Board exempted this study from review because the analysis focused on publicly available data.

The present study combined two-year survey cycles of the NHANES data from 2007–2016. Specifically, we pooled data from 2007–2008, 2009–2010, 2011–2012, 2013–14, and 2015–2016 NHANES cycle. In terms of inclusion criteria, we first selected participants that had data on biospecimens for genital HPV testing. Genital HPV testing for males was initiated in the 2013–2014 NHANES cycle, thus data prior to this cycle (i.e., NHANES 2007–2012) was restricted to only females. Second, we focused on individuals who were age-eligible for HPV vaccination (i.e., aged 12–26 years) following the vaccine’s FDA approval in 2006 and the Advisory Committee on Immunization Practices (ACIP) recommendation in 2007 (6). This resulted in the inclusion of participants who were aged 18–34 years at the time of the survey. For each survey cycle, these participants would have been within the 12–26 year eligibility window at some point prior or during the survey administration. Third, we further restricted the sample to those that provided information about their place of birth, race/ethnicity, and HPV vaccination status. The inclusion criteria were intended to capture a substantial number of foreign-born individuals in the pooled data to enable examination of HPV infection differences by nativity status. Our analytic data consisted of 4,523 participants between the ages of 18–34 years at the time of the survey administration who provided adequate sample for HPV testing. The data excludes participants who did not provide information on any of the key measures described below.

### Measures

Self-collected vaginal and penile biospecimen swabs were tested for DNA evidence of 37 HPV infection types (6, 11, 16, 18, 26, 31, 33, 35, 39, 40, 42, 45, 51, 52, 53, 54, 55, 56, 58, 59, 61, 62, 64, 66, 67, 68, 69, 70, 71, 72, 73, 81, 82, 83, 84, 89, and IS39), using the Research Use Only Linear Array Assay genotyping test (Roche Diagnostics). The main outcome measure was defined as *Any HPV Infection*, i.e., positive values for any of the 37 types tested (response option yes or no). We also examined additional measures including *Vaccine-Type Infection*, which represents positive values for 4 infection types (6, 11, 16, 18) that can be prevented with available HPV vaccination, and *High-Risk HPV Infection* (16, 18). Response options for both secondary outcomes were yes or no.

Key independent measures included *foreign-birth status*, based on what country participants were born (dichotomized as US-born versus foreign-born). Two separate race/ethnicity measures were used. The first race/ethnicity variable – categorized as Mexican, Other Hispanic, and Non-Hispanic White, Black, and Other (including multiracial groups) – is available for all survey cycles. This variable was recategorized as White, Black, Hispanic, and Other. A second race/ethnicity variable was made available starting in 2011–2012 survey cycle, which consists of Mexican, Other Hispanic, and Non-Hispanic White, Black, Asian, and Other (including multiracial groups). This measure was recategorized as White, Black, Hispanic, Asian and Other; and used in analyzing the subsample of foreign-born participants.

Covariate measures included HPV vaccination status, based on whether participant had initiated vaccination (response option yes or no), and HPV vaccination dose determined based on whether participants had received at least one dose of the HPV vaccine (yes, or no); lifetime sexual partners (categorized into 0, 1–2, and ≥ 2); and age at first sex (categorized as age <14, 14–18, and ≥19 years). Demographic variables included age, sex, education, and federal poverty level (FPL). The survey cycles were also included.

### Data analysis

**Figure 1:**
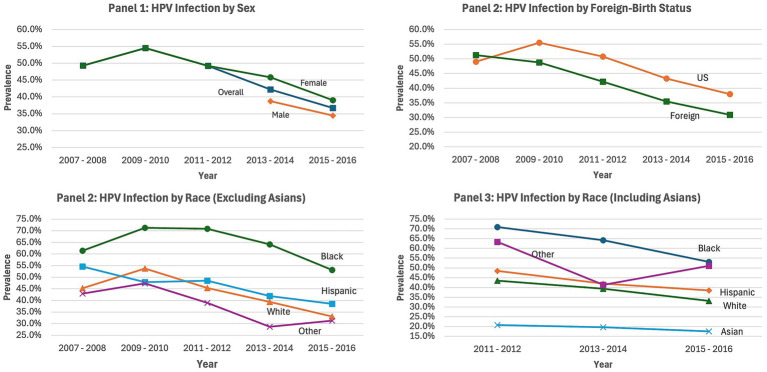
HPV infection prevalence overtime by gender, nativity, and race/ethnicity NHANES 2007–2016. Each chart depicts changes in the prevalence of HPV infection by Sex (Panel 1), Foreign-birth status (Panel 2), race/ethnicity (excluding Asian adults, Panel 3), and race/ethnicity (including Asian adults, Panel 4); NHANES, national health and nutrition examination survey.

Bivariable comparisons between foreign-birth status and HPV infection (including Any HPV, Vaccine-Type, and High-Risk Infection), demographic characteristics and other covariates were conducted using a chi-square test statistic (Table 1). HPV infection prevalence by gender, foreign-birth status, and race/ethnicity across the NHANES survey cycle were also examined (Figure 1). The foreign-born sample who provided information for the race/ethnic measure including the category ‘Asian’ (n = 871) were further examined to determine the association between race/ethnicity and any HPV infection (Table 2). First, binary logistic regression models were used to calculate the odds ratios (ORs) and 95% confidence intervals (CI) for the effect of race/ethnicity and covariates (specifically, HPV vaccination, survey cycle, age, sex, FPL, lifetime sexual partners) on any HPV infection. The covariates are known to be associated with the study outcome (20, 21). Second, a multivariable logistic regression model was used to test the relationship between race/ethnicity and HPV infection, controlling for the covariates. Third, another adjusted regression model was used to stratify this relationship by sex, due to known difference in HPV infection between males and female (12). Forward and backward selection methods were used to determine the best model fit, thus education was excluded from the models. Statistical analyses were conducted with SAS version 9.4 software. Data were weighted to account for the complex sampling methods, by following directions for using these weights for pooling data from multiple NHANES survey cycles (19).

**Table 1:** Characteristics of study sample by foreign-birth status, NHANES 2007–2016.

Variable	Overall (*n* = 4,523) *n* (%)	U.S.-born (*n* = 3,448) % (95% CI)	Foreign-born (*n* = 1,075) % (95% CI)	*P*-value
HPV outcomes				
Any HPV infection				
Yes No	2002 (43.0) 2,521 (57.0)	44.2 (41.6, 46.9) 55.8 (53.1, 58.4)	36.9 (32.8, 40.9) 63.1 (59.0, 67.1)	0.0006
Vaccine-type HPV infection				
Yes No	380 (8.2) 4,143 (91.8)	8.7 (7.6, 9.8) 91.3 (90.2, 92.4)	5.6 (3.9, 7.2) 94.4 (92.8, 96.1)	0.0025
High risk HPV infection				
Yes No	278 (6.3) 4,245 (93.7)	6.3 (5.7, 7.6) 93.4 (92.4, 94.3)	4.6 (3.1, 6.1) 95.4 (93.9, 96.9)	0.0279
Demographic characteristics				
Age				
18–26 27–34	3,031 (64.5) 1,492 (35.5)	67.0 (64.5, 69.5) 33.0 (30.5, 35.5)	52.0 (47.9, 56.2) 47.9 (43.8, 52.1)	<0.001
Sex				
Female Male	3,030 (64.8) 1,493 (35.2)	65.0 (63.0, 67.1) 34.9 (32.9, 37.0)	63.8 (59.4, 68.1) 36.2 (31.9, 40.6)	0.5814
Race/ethnicity				
White Black Hispanic Other	1,478 (56.2) 1,030 (13.7) 1,316 (20.1) 699 (10.0)	65.0 (60.9, 69.2) 14.9 (11.9, 18.0) 13.7 (11.0, 16.3) 6.4 (5.3, 7.5)	11.2 (7.6, 14.9) 7.4 (5.0, 9.8) 52.9 (45.7, 60.0) 28.5 (22.9, 34.0)	<0.0001
Married				
Married Living with partner Widowed/divorced/separated Never married	1,039 (30.7) 623 (17.3) 154 (4.1) 1803 (47.9)	28.7 (25.2, 32.2) 17.1 (14.9, 19.3) 4 (3.2, 4.8) 50.1 (46.4, 53.8)	40 (35.5, 44.5) 18.1 (14.7, 21.5) 4.5 (2.7, 6.2) 37.4 (32.5, 42.3)	<0.0001
Education				
< High school High school graduate > High school	910 (15.5) 1,138 (23) 2,474 (61.5)	13.3 (11.7, 14.9) 23.6 (60.2, 66.0) 63.1 (60.2, 66.0)	26.5 (21.3, 31.7) 19.9 (16.5, 23.22) 53.6 (48.4, 58.9)	<0.0001
FPL (%)				
< 100 100 to <200 200+	1,326 (24.1) 1,085 (24.0) 1740 (51.9)	22.4 (19.9, 24.9) 23.7 (21.8, 25.6) 53.9 (50.7, 57.0)	33.1 (28.6, 37.5) 25.5 (22.2, 28.8) 41.4 (36.3, 46.5)	<0.0001
Health behavioral factors				
HPV vaccination				
Yes No	991 (75.9) 3,170 (24.1)	26.1 (23.9, 28.4) 73.9 (71.6, 76.1)	13.4 (10.2, 16.6) 86.6 (83.4, 89.8)	<0.0001
HPV vaccination dose				
1 2 3	181 (17.4) 178 (18.6) 558 (64.0)	17.1 (13.1, 21.1) 18.1 (15.0, 21.2) 64.8 (59.7, 69.9)	21.3 (12.3, 30.2) 24.6 (16.5, 32.7) 54.1 (44.1, 64.2)	0.1264
Age at first sex (years)				
<14 14–18 19+	359 (8.6) 2,541 (69.9) 724 (21.5)	9.4 (7.9, 10.8) 71.7 (69,3, 74.1) 18.9 (16.5, 21.3)	4.1 (2.6, 5.6) 59.9 (55.8, 64.0) 36.0 (31.5, 40.5)	<0.0001
Lifetime sexual partners				
0 1–2 2+	582 (13.3) 1,030 (24.3) 2,386 (62.4)	13.3 (11.8, 14.7) 22.3 (19.9, 24.6) 64.5 (61.8, 67.1)	13.1 (10.9, 15.4) 35.7 (31.8, 39.6) 51.2 (47.1, 55.3)	<0.0001

**Note.** Abbreviations: HPV, Human Papillomavirus; FPL, Federal Poverty Level; NHANES, National Health and Nutrition Examination Survey. P-values were calculated using Chi-square tests; n, represents raw frequencies; all percentages are weighted. “HPV vaccination,” refers to those who received at least one does of HPV vaccination. “Race/ethnicity,” available for all survey cycles with original categories including Mexican, Other Hispanic, and Non-Hispanic White, Black, and Other. NHANES 2007-2012 data was restricted to only females, but NHANES 2013-2016 data included both males and females since Genital HPV testing for males began with the 2013-2014 NHANES cycle.

**Table 2:** Adjusted odds of HPV infection by race/ethnicity among foreign-born adults, stratified by sex, NHANES 2011–2016 (*N* = 871).

	FB total (*N* = 871)		FB female (*n* = 504)	FB male (*n* = 367)
	Model 1	Model 2	Model 3	Model 4
	OR (95% CI)	aOR (95% CI)	aOR (95% CI)	aOR (95% CI)
Race/ethnicity				
White Black Hispanic Asian Other	Ref **3.01 (1.45, 6.19)** 1.66 (0.79, 3.52) **0.40 (0.18, 0.87)** 1.46 (0.47, 4.50)	Ref **3.38 (1.3, 8.6)** **2.24 (1.1, 4.6)** 0.54 (0.3, 1.1) 2.02 (0.5, 7.8)	Ref **5.27 (1.51, 18.41)** **2.99 (1.26, 7.11)** 0.53 (0.21, 1.32) 1.97 (0.49, 7.83)	Ref 2.06 (0.24, 17.92) 2.62 (0.24, 11.03) 0.60 (0.11, 3.27) 2.51 (0.15, 42.43)
HPV vaccination				
No Yes	Ref 1.47 (0.99, 2.20)	Ref 1.51 (0.9, 2.7)	Ref 1.64 (0.72, 3.69)	Ref 0.97 (0.32, 2.934)
Survey cycle				
2011 2013 2015	Ref 0.75 (0.46, 1.24) 0.61 (0.36, 1.05)	Ref 0.82 (0.4, 1.7) 0.41 (0.2, 0.8)	Ref 0.72 (0.35, 1.49) **0.46 (0.22, 0.93)**	Ref **6.11 (1.45, 25.72)** **--**
Age	0.98 (0.94, 1.01)	1.00 (0.9, 1.1)	0.96 (0.88, 1.05)	1.05 (0.97, 1.14)
Sex				
Female Male	Ref **0.71 (0.52, 0.97)**	Ref 0.87 (0.6, 1.4)	--	--
FPL				
<100 100 to <200 200+	Ref 0.80 (0.52, 1.23) 0.70 (0.46, 1.07)	0.72 (0.4, 1.3) 0.80 (0.4, 1.5)	0.90 (0.47, 1.72) 1.01 (0.45, 2.25)	Ref 0.54 (0.21, 1.38) 0.56 (0.20, 1.52)
Lifetime sexual partners				
0 1–2 2+	Ref 0.79 (0.46, 1.35) **3.27 (1.95, 5.50)**	Ref 1.35 (0.4, 4.6) **5.95 (2.0, 17.5)**	Ref 0.85 (0.23, 3.11) **3.77 (1.17, 12.09)**	Ref 3.20 (0.49, 20.95) **13.22 (2.51, 69.65)**

**Note.** Abbreviations: FB, Foreign Born; HPV, Human Papillomavirus; FPL, Federal Poverty Level; NHANES, National Health and Nutrition Examination Survey; OR, Adjusted Odds Ratio; aOR, Adjusted Odds Ratio. Bolded ORs and aORs (95% CI) represents significant findings at p< 0.05; Ref, Referent Group; ”--”, Not estimated due to small cell size. The race/ethnicity variable used to assess the subsample of foreign-born participants was made available starting in 2011-2012 survey cycle, with original categories including Mexican, Other Hispanic, and Non-Hispanic White, Black, Asian, and Other (including multiracial groups).

## Results

HPV infection in the overall sample was 43%, and HPV vaccine-type infection was 8% (Table 1). Approximately 76% of all participants had initiated receipt of HPV vaccination. A difference in HPV infection (44% vs. 37%, *p* < 0.001), vaccine-type infection (9% vs. 6%, *p* < 0.01), and HPV vaccination initiation (26% vs. 13%, *p* < 001), was observed between US- and foreign-born participants, respectively. For the demographics, over half of the total sample was female (65%), age 18–26 years (65%), and White (56%); and under half were married (31%). A majority of all participants reported having at least some post-high school education (62%) and a little over half (52%) reported incomes 200% above the federal poverty level. In terms of covariates, most participants reported initiation of sexual intercourse between age 14–18 years (70%) and having at least 2 lifetime sexual partners (62.4%). Most of the described factors, except for sex and HPV vaccination dose, differed between U.S.- and foreign-born participants (*p* < 0.001).

HPV infection prevalence overtime between 2007–2016 by sex, foreign-birth status, and race/ethnicity are presented in Figure 1. For Panel 1 (HPV infection prevalence by sex), HPV infection for females decreased across cycles following the 2009–2010 survey cycle. HPV infection data collection for males was initiated in the 2013–2014 cycle, and there was an observed decrease in infection for males in the 2015–2016 survey cycle. Panel 2 (HPV infection prevalence by foreign-birth status) revealed that after an increase in HPV infection between the 2007–2008 and 2009–2010 survey cycles among US-born participants, the infection rates decreased in subsequent cycles. For foreign-born participants, the infection rates decreased following the 2007–2008 cycle. In Panel 3 (HPV infection prevalence by race), examination of race/ethnicity, excluding Asian populations, indicated that HPV infection was highest among Black adults (61%) followed by Hispanic adults (55%) and White adults (45%) in the 2007–2008 survey cycle. There was an overall decrease in HPV infection for each race/ethnicity across the survey cycles, following the 2009–2008 cycle, with a significant difference in the proportion of HPV infection among Black, Hispanic, and White adults (53% vs. 39% vs. 33%, *p* < 0.001) in the 2015–2016 survey cycle. For Panel 4 (HPV infection prevalence by race), assessment of race/ethnicity including Asian adults was possible for the survey cycles between 2011–2016. This revealed that HPV infection was highest for Black adults and lowest for Asian adults from the 2011-2012 survey cycle (71% vs. 21%, *p* < 0.001) through the 2015-2016 cycle (53% vs. 17%, *p* < 0.001). The percent decrease in HPV infection across cycles (between 2011 and 2015) was biggest for Black adults (17%), followed by White adults (12%), and lowest for Asian adults (3%), *p* < 0.001.

Regression models were run for the foreign-born sample to determine the association between race/ethnicity and any HPV infection (Table 2). Model 1, the unadjusted model, revealed that among all foreign-born participants, the odds of HPV infection were significantly higher for Black adults than White adults (OR, 3.01; 95% CI, 1.45–6.19). Asian adults had significantly lower odds of HPV infection in comparison to White participants (OR, 0.40; 95% CI, 0.18–0.87). Additionally, foreign-born males also had significantly lower odds of HPV infection than foreign-born females (OR 0.71; 95% CI, 0.52–0.97). In Model 2, the adjusted model for all foreign-born participants, the higher odds of HPV infection among Black adults remained (aOR, 3.38; 95% CI, 1.3–8.6). Hispanic participants had significantly higher odds of HPV infection than White participants (aOR, 2.24; 95% CI, 1.1–4.6). Also, foreign-born participants who reported 2 + lifetime sexual partners versus no sexual partners were significantly more likely to report HPV infection (aOR, 5.95; 95% CI, 2.0–17.5).

The adjusted regression model was further stratified by sex (Table 2). In Model 3, among foreign-born females, HPV infections were significantly higher for Black (aOR, 5.27; 95% CI, 1.51–18.41) and Hispanic participants (aOR 2.99; 95% CI, 1.26–7.11) as compared to White participants. Also, foreign-born females with at least two lifetime sexual partners had 3.77 greater odds of reported HPV infection than those with no sexual partners (95% CI, 1.17, 12.09). For Model 4, among foreign-born males, race was not associated with HPV infection. Those with at least two lifetime sexual partners had significantly higher odds of HPV infection (aOR 13.22; 95% CI 2.51–69.65).

## Discussion

In this study we found an overall decline in HPV infection across the 2007–2016 survey cycles, by foreign-born status, sex, and race/ethnicity. Besides the decreasing HPV infection prevalence overtime among race/ethnic groups, Black adults reported the highest rates of infection across cycles, followed by Hispanic adults; and Asian adults reported the lowest rates. Further examination of the foreign-born sample revealed that both foreign-born Black and Hispanic adults had a higher odds of HPV infection after controlling for covariates. These associations remained significant for females but not males.

Prior research supports the findings that HPV infections, including vaccine type infections, have decreased among both females and males (22, 23). This is largely associated with the availability of HPV vaccination (23). We note that while we did not find any significant sex differences in HPV infection in our sample, earlier studies suggest that men have a higher rate of HPV infections than women (12, 13). The NHANES data collection of HPV infection among males was initiated in the 2013–2014 cycle, which limits our ability to make nuanced comparisons between males and females. Other researchers, however, argued that a decrease in HPV infections among males could be related to their uptake of HPV vaccines as well as the indirect association of decreasing transmission from females (2, 24).

Consistent with prior literature, we observed disparities in HPV infections among racial/ethnic groups and by foreign-birth status. For example, examination of general, as well as oral, HPV infections among adults 18–69 years using the NHANES data revealed the highest rate among Black adults and lowest among Asian adults (12, 13). The disproportionate HPV infection rates among Black adults remained after adjusting for known factors associated with infection including gender, lifetime sexual partners, and substance use (12). Our findings also contribute to the limited studies that have found that HPV infection rates were significantly lower in foreign-born compared with US-born individuals (14, 15). Shahmoradi et al. (15) recently examined the relationship between HPV vaccination and HPV infection by estimating the rates of HPV infection types 16 and 18 (which are covered by 4-valent vaccine) before and after the vaccine introduction in the US. They found that US-born individuals consistently reported higher rates of HPV infection than the foreign-born – among cohorts born in the 1980s and 1990s and among adults in the pre-vaccination (2005–2006 survey cycle) and recent (2015–2016 survey cycle) periods.

The present study contributes new evidence of race/ethnic differences in HPV infection among foreign-born individuals. Particularly, foreign-born Black and Hispanic adults had a three-fold and two-fold increase in their odds having HPV-infections, respectively, compared with foreign-born White adults, after adjusting for factors including number of life-time sexual partners, HPV vaccine status, and sex. This finding has wider implications on the negative health consequences of HPV infections among foreign-born individuals. For instance, current research suggests that both cervical cancer incidence and morbidity remain higher in minorities such as U.S. Black and Hispanic/Latina women. The mortality rate of cervical cancer in Black women is significantly higher in comparison with White women (25). Studies using national level health data often group US- and foreign-born individuals together. However, recent evidence indicated that whereas the cervical cancer mortality rate for foreign-born women is lower than the US-born, death rates are rising among older foreign-born women (26). A possible explanation is that length of residency in the U.S. is likely associated with an increased risk of HPV infection among foreign-born individuals. HPV vaccines, which are officially recommended and available in the U.S. are not currently required for immigration (27). Moreover, challenges to navigating the U.S. healthcare system compounds barriers to HPV vaccine uptake among foreign-born individuals. Thus, lower uptake of HPV vaccine among foreign-born individuals may greatly increases the risks of developing HPV-associated cancers.

Given the high rates of HPV infection among race/ethnic minority U.S. immigrants, future intervention efforts may consider known barriers to HPV vaccine uptake in this group. These barriers include limited English proficiency, limited HPV-related knowledge, healthcare barriers such as lack of insurance, and structural barriers such as cultural or religious bias against HPV vaccination (18, 28–30). Future programs may explore the use behavioral change theories (e.g., health belief model, theory of planned behavior, and social cognitive theory) to help foreign-born individuals dispel their concerns about HPV vaccines and take the necessary to receive the vaccine, as well as social determinants of health based interventions to increase access to healthcare. For instance, HPV vaccination uptake among foreign-born individuals can be significantly increased by implementing efforts to improve their level of education and English language proficiency (31). Also, with the age of eligibility for HPV vaccine extended to 45 years, many foreign-born individuals are eligible for HPV vaccination upon immigration. A potential area for further exploration could be the review and update the immigration immunization policy.

### Limitations

A strength of this study includes the use of a sample representation of the U.S. adult population, containing a significant proportion of foreign-born individuals. Moreover, medical examination data enabled U.S. to determine HPV infection rates among study participants. However, there were a few limitations. First, the study’s HPV vaccination and other health related survey questions were based on self-reported data, and thus responses provided by participants may be subject to recall bias. Second, given that data collection for the NHANES is based on a cross-sectional design, it is impossible to draw causal inference from the findings, nor to confirm of whether vaccination preceded infection. The stratified analyses for some racial/ethnic groups may be underpowered. Also, estimates with wide CIs should be interpreted with caution due to potential model instability.

## Conclusion

The present study is one of the few studies to use NHANES data to examine HPV infection differences among foreign-born adults. The findings suggest lower HPV infection, vaccine-type infection, and vaccination initiation rates among foreign-born adults. Among foreign-born adults, HPV infection was higher among Black individuals; and among foreign-born females, HPV infection were higher among Black and Hispanic individuals. While these findings are based on cross-sectional data, and not causal relationships, they may inform public health interventions to reduce HPV infection risks and promote HPV vaccination initiation among foreign-born Black and Hispanic individuals. Moreover, further research may be implemented to explore additional risk factors associated with HPV infection difference by nativity and sex.

## Data Availability

The original contributions presented in the study are included in the article/supplementary material, further inquiries can be directed to the corresponding author/s.
